# Graphene liquid crystal retarded percolation for new high-*k* materials

**DOI:** 10.1038/ncomms9700

**Published:** 2015-11-16

**Authors:** Jinkai Yuan, Alan Luna, Wilfrid Neri, Cécile Zakri, Tanja Schilling, Annie Colin, Philippe Poulin

**Affiliations:** 1 Centre de Recherche Paul Pascal, CNRS, Université de Bordeaux, 115 Avenue Schweitzer, 33600 Pessac, France; 2 University of Bordeaux, CNRS, Solvay, LOF, UMR 5258, 33608 Pessac, France; 3 Physics and Materials Science Research Unit, Université du Luxembourg, 162A, Avenue de la Faïencerie, L-1511 Luxembourg

## Abstract

Graphene flakes with giant shape anisotropy are extensively used to establish connectedness electrical percolation in various heterogeneous systems. However, the percolation behaviour of graphene flakes has been recently predicted to be far more complicated than generally anticipated on the basis of excluded volume arguments. Here we confirm experimentally that graphene flakes self-assemble into nematic liquid crystals below the onset of percolation. The competition of percolation and liquid crystal transition provides a new route towards high-*k* materials. Indeed, near-percolated liquid-crystalline graphene-based composites display unprecedented dielectric properties with a dielectric constant improved by 260-fold increase as compared with the polymer matrix, while maintaining the loss tangent as low as 0.4. This performance is shown to depend on the structure of monodomains of graphene liquid-crystalline phases. Insights into how the liquid crystal phase transition interferes with percolation transition and thus alters the dielectric constant are discussed.

## Abstract (web summary)

It is commonly believed that graphene flakes form electrical percolation networks at low concentration, and thus can be used as conductive materials. Here, Yuan *et al*. show in graphene polymer composites that the transition to liquid crystals hinders the formation of percolated networks, resulting in high-*k* materials.

## Graphical abstract

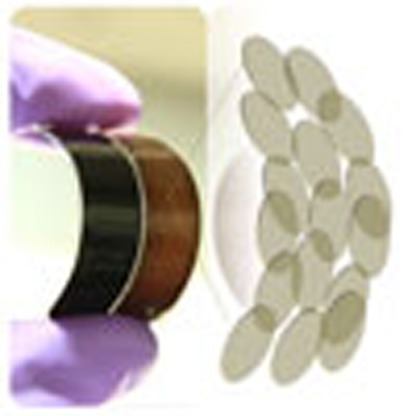

The concept of percolation, initially discussed in the context of scaling behaviour and universality in statistical physics, finds today a variety of applications in materials research as a powerful framework to account for the physical properties of heterogeneous materials^1^. The significance of the percolation transition lies in the fact that the global connectivity of the minor phase (for example, conducting fillers in an insulating medium) at the percolation threshold immediately results in a dramatic change in the transport properties (for example, electrical or thermal conductivity and diffusion) instead of following a linear rule of mixtures^2^,^3^. Such nonlinear scaling is exploited below percolation, to improve other properties such as dielectric constant^4^,^5^,^6^ or optical nonlinearity^7^. Technological applications of this concept generally demand a low percolation threshold.

Percolation at very low concentrations has often been found in systems with rod-like particles of large aspect ratios in the macroscopically isotropic state^8^. In addition to the connectedness percolation transition, on increasing concentration rod solutions also undergo an isotropic–nematic transition, which breaks the rotational symmetry^9^. The location of the percolation threshold depends on the connectivity length, that is, the surface-to-surface distance below which two rods are in contact, whereas the location of the isotropic–nematic transition is independent of this length. Thus, in principle, the order in which the two transitions occur when one increases the concentration of the rods depends on the connectivity length. However, when taking realistic electron tunnelling lengths as the connectivity length, the percolation transition in rods always occurs at a concentration far below the isotropic–nematic transition. When extended to impenetrable platelets, the situation can be different. It has been recently predicted that the isotropic–nematic transition could occur below the onset of percolation expected from excluded volume arguments of randomly oriented platelets^10^. If true, this phenomenon would significantly change our views on the interest of using graphene for making conductive nanocomposites^11^. The competition of the percolation transition with transitions to liquid crystals (LCs) near thermal equilibrium could actually hinder the formation of conductive networks, making the common belief that graphene flakes exhibit a low percolation threshold erroneous. This statement is supported by simulations and it can explain the large variability of experimental results in the literature with percolation thresholds varying from a fraction of vol% to tens of vol%. The very low values do not reflect actual statistical percolation but out of equilibrium gelation mechanisms.

The absence of percolation in equilibrated samples could still be exploited positively from a technological point of view, in particular for the development of new dielectric materials. There is a fast growing interest in developing graphene-containing composites that bear favourable dielectric properties^12^,^13^,^14^. They can potentially serve as high-*k* materials for various applications, including gate dielectrics, energy storage devices and electroactive materials^12^,^13^,^14^.

However, experimental identification of the manifestation of competing percolation and LC phase transition requires the challenging development of graphene suspensions that can percolate or form LCs at rest without being affected by non-equilibrium mechanisms such as flow or volume changes in drying systems. Liquid crystallinity of monolayer graphene in aqueous media^15^,^16^ or polar organic solvents^17^,^18^ has been demonstrated and even already used to prepare composite fibres^19^,^20^ or dielectric composites^21^. To date, however, extensions of their applications to hydrophobic polymer composites still remain limited because of the difficulty of forming graphene LCs in non-polar organic solvents at thermal equilibrium.

In this work, we have succeeded in producing LCs of graphene oxide (GO) in a non-polar organic solvent. Such suspensions can readily serve as precursors to prepare polymer composites with polydimethylsiloxane (PDMS) as matrix. This unique system allows the demonstration that large graphene flakes spontaneously form LCs before they percolate, confirming therefore the theoretical prediction that graphene flakes, in spite of their giant aspect ratio, do not exhibit a low percolation threshold. In addition to providing the first test of this prediction, the present materials have unique dielectric properties. They exhibit a very high dielectric constant of 753 at 100 Hz along with a low loss tangent of only 0.4. These findings are generic and expected in other composites manufactured from dispersions of highly conductive platelets with large aspect ratios, opening thereby a new route towards efficient high-*k* materials.

## Results

### Phase transfer of GO from the aqueous to organic phase

**Figure 1:**
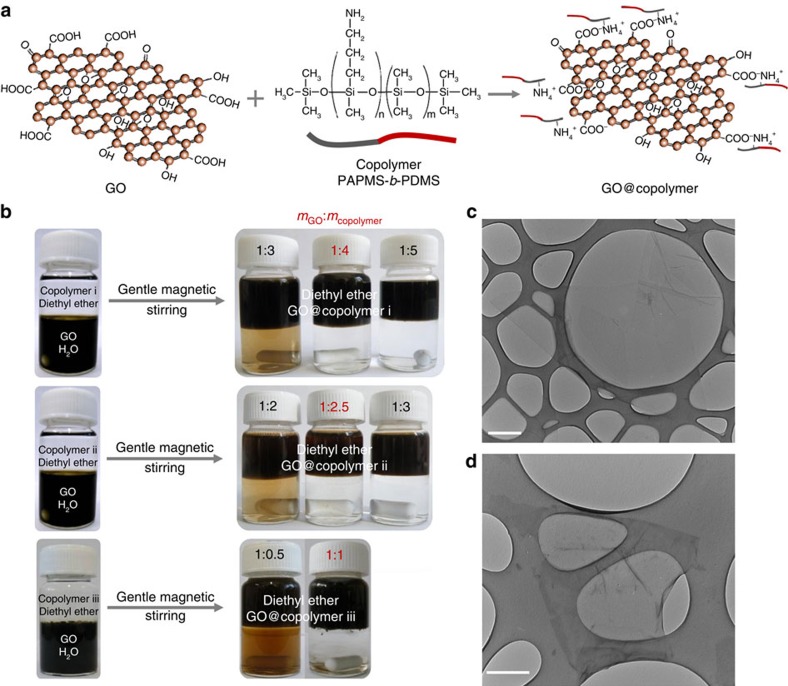
Phase transfer of GO from the aqueous to the organic phase. (**a**) Schematic illustration of the electrostatic attraction between negative charges of GO nanosheets and positive ones of the PAPMS-*b*-PDMS copolymer. (**b**) Photographs show the phase transfer of GO flakes from the aqueous to the organic phase by using PAPMS-*b*-PDMS copolymers with different mole concentrations of amine groups. The starting volumes of the oil and water phases are 10 ml. The amount of GO in the starting aqueous phase is 1 mg ml^−1^. The phase transfers are shown as a function of the weight ratio of GO to copolymer (*m*_GO_:*m*_copolymer_). It is considered as completed when the lower aqueous phase is uncoloured. (**c**) TEM image showing a flake of pristine GO. (**d**) TEM image of GO flakes after functionalization with copolymer i. The samples for TEM characterization are prepared by depositing a drop of sufficiently diluted pristine GO aqueous solution or modified GO organic solution onto standard TEM grids. (**c**,**d**) Scale bars, 500 nm.

Processing GO in organic solvents is not straightforward, as it needs modifying its surfaces with adequate chemical functionalities^22^. Previous state-of-the-art methods used to functionalize GO sheets necessitate anhydrous conditions and harsh reagents^23^,^24^. Here we functionalized GO flakes with a diblock copolymer polyaminopropylmethylsiloxane-*b*-PDMS copolymer (PAPMS-*b*-PDMS) under ambient conditions via electrostatic attractions (Fig. 1a). The method is easily scalable (Supplementary Fig. 1) and in particular addresses the limitations of other methods for GO modification, such as high reaction temperatures or irreversible covalent bonding. Positively charged amine functions of the PAPMS segments electrostatically interact with the negatively charged carboxylic groups of the GO flakes (Fig. 1a). Copolymers and GO flakes self-assemble under gentle stirring at the oil/water interface of a biphasic mixture composed of copolymer solution in diethyl ether as top phase and a GO aqueous solution as bottom phase. The copolymer attracts GO nanosheets from the aqueous to the organic phase and eventually stabilizes their dispersion in diethyl ether (Fig. 1b). The copolymer-derivatized GO flakes are no longer soluble in water but readily form stable dispersions in non-polar organic solvents by pointing hydrophobic PDMS blocks towards the organic phase. It should be noted that the underlying phase transfer mechanism is pH sensitive. A pH of the aqueous solution typically between 4 and 10 is required so that the amine and carboxylic groups remain, respectively, positively and negatively charged.

**Figure 2:**
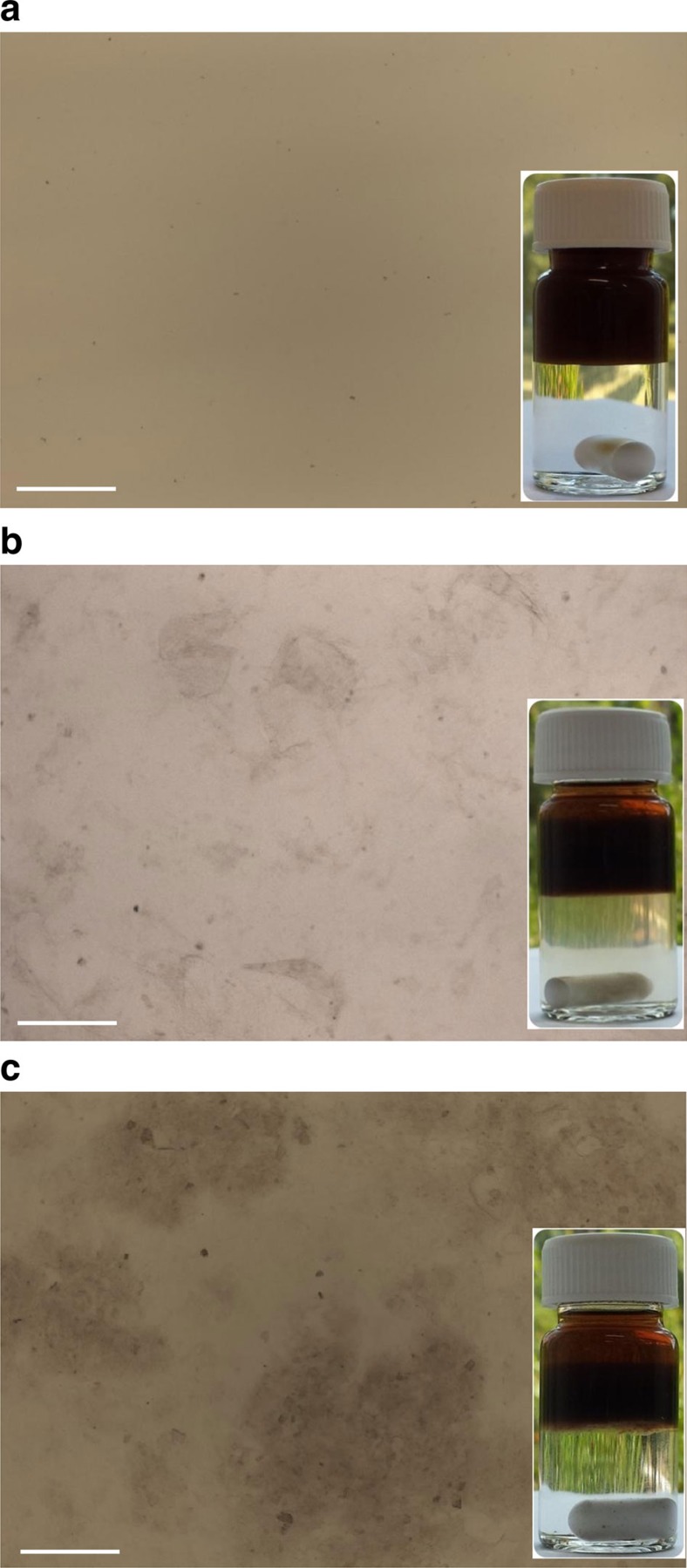
Optical micrographs of GO suspensions in diethyl ether. (**a**) Dispersions stabilized by copolymer i (*m*_GO_:*m*_copolymer i_=1:4) are free of aggregates. By contrast, aggregates can be observed for copolymers ii (**b**) and iii (**c**) stabilized GO suspensions, which are achieved with the mass ratio of *m*_GO_:*m*_copolymer ii_=1:2.5 and *m*_GO_:*m*_copolymer iii_=1:1, respectively. (**a**–**c**) Scale bars, 100 μm; insets show images of macroscopic vials after 2 days. Sedimentation of aggregates can be seen in the upper organic phases for copolymer ii (**b**) and iii (**c**). Suspensions stabilized by copolymer i (**a**) remain stable.

Three PAPMS-*b*-PDMS copolymers named i, ii and iii were tested. These materials are under the form of isotropic liquids at room temperature. As shown in Fig. 1b, less copolymer is needed to fully extract the GO sheets for copolymers ii and iii. However, these copolymers are found in fact to be less efficient at stabilizing GO against aggregation than copolymer i in the organic phase. As shown in Fig. 2, aggregates in the organic phase are observed by optical microscopy in the cases of dispersions stabilized by copolymer ii or by copolymer iii. This is not the case for copolymer i. Sedimentation of the aggregates is observed on macroscopic scale after a few days (insets in Fig. 2). This behaviour reflects that the most hydrophobic copolymer i provides an efficient steric repulsion against aggregation. Therefore, copolymer i was chosen to modify the GO flakes and the copolymer i-derivatized GO (GO@copolymer) nanosheets were further incorporated into a PDMS matrix to form polymer composites.

The surface morphologies of GO and GO@copolymer flakes were further investigated by transmission electron microscopy (TEM). As shown in Fig. 1c, the pristine GO monolayer with a sheet dimension of 2–3 μm is highly electron transparent, as the copper mesh is clearly visible through the flake. After functionalization with copolymer i, one can observe that the GO@copolymer particle still exists as a very thin monolayer with similar sheet dimension to pristine GO (Fig. 1d). These results confirm that functionalization by PAPMS-*b*-PDMS does not degrade the graphene flakes. Raman spectral analyses further clarify the interaction between copolymer and GO flakes (Supplementary Fig. 2). As compared with pristine GO, GO@copolymer particles have an unchanged G band, yet a blue-shifted D band, indicating that the copolymer does not modify the *sp*^2^ carbon lattice structure but essentially interacts with functional groups of the GO sheets.

### Liquid-crystalline graphene-based polymer composites

**Figure 3:**
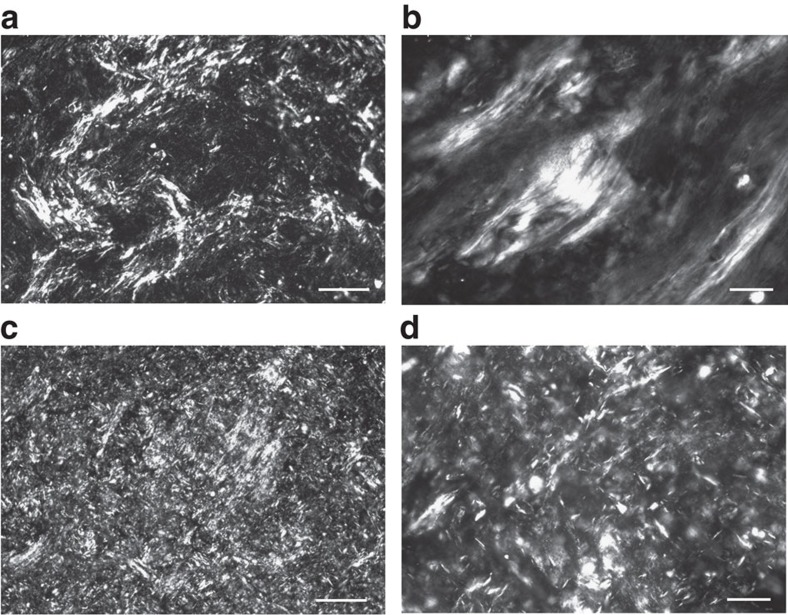
LC phases of GO in non-polar suspending media. (**a**,**b**) Low-magnification (**a**) and high-magnification (**b**) optical micrographs between crossed polarizers of GO suspensions in diethyl ether at a concentration of 1 wt%. (**c**,**d**) Low-magnification (**c**) and high-magnification (**d**) micrographs of viscous GO/PDMS composite precursors with 1 wt% of GO before thermal cross-linking. (**a**,**c**) Scale bars, 100 μm. (**b**,**d**) Scale bars, 20 μm. Samples were prepared by transferring a drop of GO suspension onto a glass slide and confining the dispersion with a cover slip. For the GO/PDMS sample, the GO/PDMS suspensions were first dried in the vacuum oven at room temperature for 15 min and then the viscous mixture was transferred onto a glass slide and confined with similar cover slip. The observed textures reflect the formation of small monodomains of nematic LC phase.

The present method provides a route towards highly concentrated suspensions of GO in non-polar organic solvents. It could in principle lead to the formation of LCs on increasing concentration, as a consequence of the large aspect ratio of the GO flakes. To test this possibility, we have characterized GO@copolymer directly after their phase transfer at a concentration of about 1 mg ml^−1^ by optical microscopy under crossed polarizers. At this concentration, the suspensions are found to be isotropic and appear homogeneous and black. However, more concentrated samples controlled by partial evaporation of the solvent display optical birefringence. The latter reveals a liquid-crystalline order (Fig. 3a,b) and is observed for weight fractions as low as 1 wt%. Such LCs made of platelets are often called discotic LCs. Orientational ordering revealed by optical birefringence is maintained as the concentration is increased further by evaporating the highly volatile diethyl ether solvent. The LCs are made of small and randomly oriented monodomains. The present method can be extended to other solvents by adding a fluid that is less volatile but miscible with diethyl ether. In particular, it can be extended to pure PAPMS-*b*-PDMS used as solvent or PDMS precursors, which are well soluble in diethyl ether. All these systems display similar birefringent textures when observed under optical microscopy between crossed polarizers. More quantitative characterization of the liquid-crystalline order has been achieved using small-angle X-ray scattering (Supplementary Fig. 3). These experiments have been performed for the binary mixtures of GO and pure PAPMS-*b*-PDMS, to characterize positional ordering. As sketched in Supplementary Fig. 4, spatial correlations are pronounced in concentrated materials but become weak for more dilute samples. The effective thickness of the GO platelets, which accounts for the actual thickness of the particles and their roughness and undulations, is found to be of about 0.85 nm. The structure evolution as a function of concentration is well consistent with the expectation that the particles are stabilized by steric repulsions provided by absorbed PAPMS-*b*-PDMS copolymers (Supplementary Figs 3 and 4). GO/PDMS composite precursors can be directly made by the phase transfer in the presence of PDMS precursors in the upper organic phase. After complete evaporation of the liquid medium, the graphene flakes remain suspended in the fluid solely comprising PDMS precursors. The concentration can be controlled by varying the amount of added PDMS precursors. Similar to their parent GO suspensions in diethyl ether, this composite formulation displays birefringence (Fig. 3c,d). It is noteworthy that the general texture and size of the monodomains depend on the samples. GO LCs in PDMS are more viscous than GO LCs in diethyl ether and exhibit smaller monodomains and numerous topological defects. The greater amount of defects can be explained by their slower coarsening in the more viscous materials^25^,^26^,^27^,^28^.

**Figure 4:**
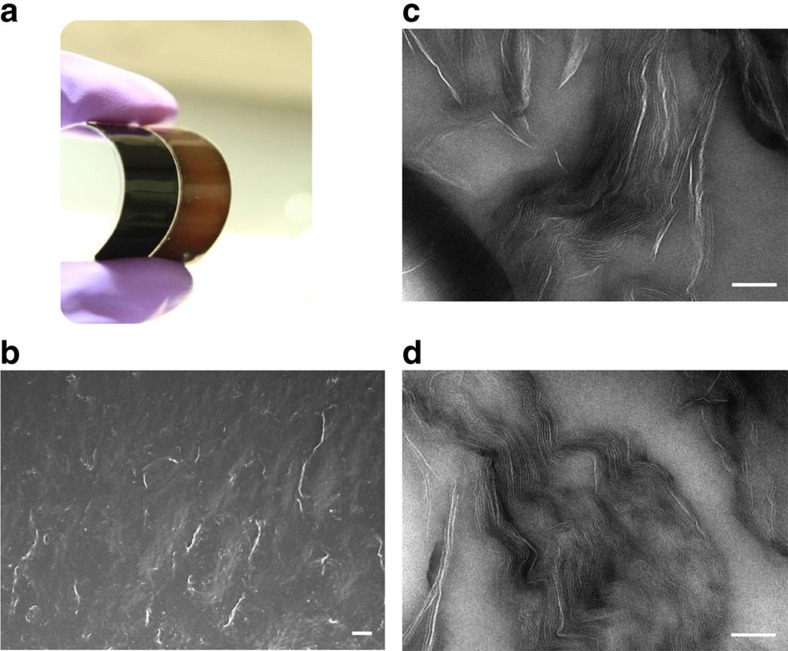
LC graphene-based PDMS composites. (**a**) Photograph of soft PDMS composites with GO (brown) and rGO (black) nanosheets at a loading of 1 wt%. (**b**) SEM image of the fractured surface of a rGO/PDMS composite with a nanosheet content of 1 wt%. Scale bar, 10 μm. Cross-section of composites was obtained by immersion of the composite in liquid nitrogen and breaking the materials. (**c**,**d**) TEM images of GO (**c**) and rGO (**d**) flakes in PDMS composite with a loading of 1 wt%. Scale bars, 100 nm. Aligned structures resultant from the liquid crystallinity of the parent materials are observed.

Owing to the use of the liquid and cross-linkable precursors as suspending medium, GO liquid crystallinity is well maintained throughout the all processing, from the parent equilibrated phase until its solidified state. The present systems are ideal models to explore the manifestation of competing percolation and LC phase transition. To electrically assess connectivity percolation, the obtained GO/PDMS composites need to be thermally reduced to restore the electrical conductivity of the GO flakes (Fig. 4a). The dispersion state of reduced GO (rGO) nanosheets was examined and visualized in scanning electron microscopy (SEM) images of fractured surfaces of rGO/PDMS composites (Fig. 4b). In addition, TEM images of GO flakes in nanocomposites, as shown in Fig. 4c,d, show locally aligned morphologies due to the formation of small and randomly oriented monodomains of LCs. The textures before and after thermal reduction are similar. This observation indicates that the thermal treatment does not affect the structure of the materials.

### Isotropic-nematic transition versus percolation

It has been conjectured in the 1980s that the percolation threshold of anisotropic objects scales as the ratio of the volume of one particle to the excluded volume between two particles^29^. In the case of rods of diameter *D* and length *L*, as well as platelets of thickness *D* and diameter *L*, the percolation threshold would thus be expected to scale as the inverse of the aspect ratio,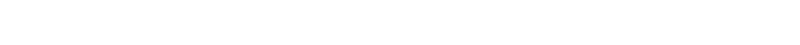 
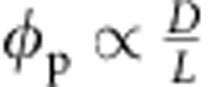
 (ref. 30). However, the percolation behaviour of anisotropic particles can be subtle and often counterintuitive. The clustering mechanism is affected by polydispersity^31^,^32^, interactions between the particles^3^,^33^, correlation of orientations^34^ and bundling of rods or stacking of platelets^35^. Deviations from the excluded volume scaling are expected to occur, in particular when the percolation transition competes with the transition to liquid-crystalline phases^10^,^36^.

Recent simulations of impenetrable platelets have shown that the percolation threshold does not follow scaling with the inverse aspect ratio^10^. On increasing concentration, hard particles (rods and platelets) of large aspect ratio preferentially align with each other to maximize packing entropy rather than forming percolated networks of randomly oriented particles. Thus, there is a competition between percolation and nematic ordering. Which of the two transitions occurs first, that is, at a lower concentration than the other, depends on the shape of the particle and on an additional characteristic length, the connectivity length. This length is the typical length over which charge carriers have to tunnel from one particle to another. For realistic tunnelling lengths, the percolation threshold in suspensions of rod-like particles is always below the isotropic–nematic transition. In contrast, in suspensions of platelets, the isotropic–nematic transition can occur at lower concentrations than the percolation transition and thus suppress the formation of a conducting network. For large aspect ratios, the percolation threshold then becomes almost independent of the aspect ratio of the platelets. In simulations of hard-cut spheres^10^ we found a constant percolation threshold at *D*/*L*>60 for a connectedness length equal to the particle thickness *D*. The onset of the plateau shifts to smaller aspect ratios with decreasing connectedness length. Thus, for any realistic connectedness length, platelets of aspect ratios above 100 should be in the regime of a constant percolation threshold. We rationalized this unusual independence of the percolation threshold from the aspect ratio in the following way: we employed the excluded volume argument given above, but instead of randomly oriented disks we used the volume of oriented disks as predicted by an effective single-particle cell model. The cell model reproduced both the nematic-order parameter and the percolation threshold quite accurately. For details, please see ref. 10.

**Figure 5:**
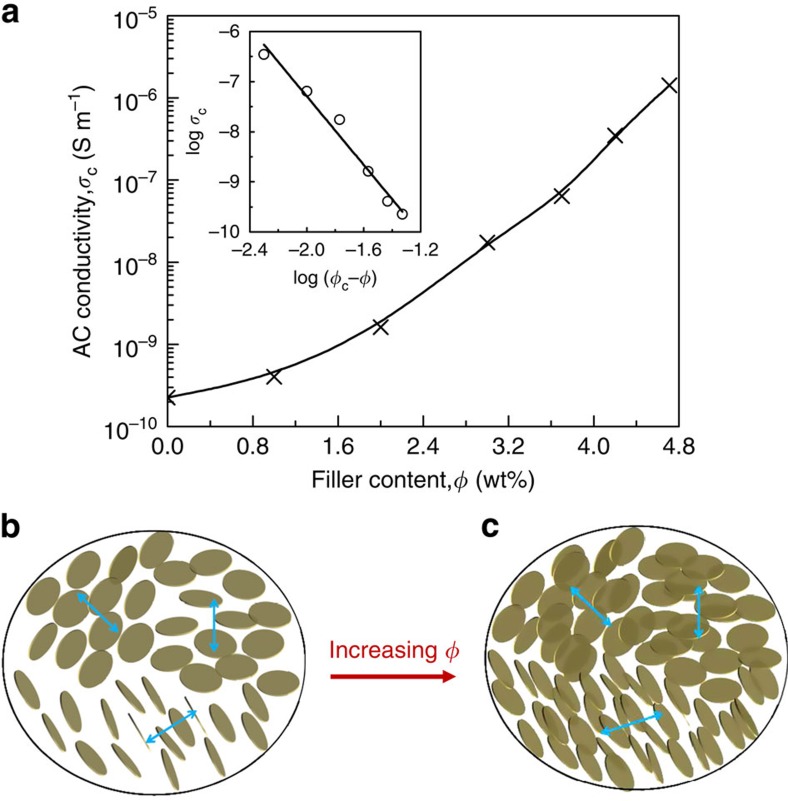
Interference of LC transition with percolation transition. (**a**) AC conductivity of the rGO/PDMS composites as a function of the filler content, measured at room temperature and 100 Hz. Inset shows the best fit of the conductivity to a power law, which gives rise to a percolation threshold *ϕ*_c_=4.7 wt% and a critical exponent *s*=3.42. It is worth noting that, except for the first point in which no rGO is present, all the characterized materials display a liquid-crystalline texture. (**b**) Microstructural representation of PDMS composites, which are composed of randomly oriented monodomains of LCs at low concentration. In each domain, the flakes tend to be aligned but no percolating path is established. (**c**) Schematic illustration of the microstructure of the composite at high concentration. In this case, the monodomains still remain randomly orientated but flakes inside domains begin to percolate and eventually form an infinity network of connected path through the insulating matrix.

Experimentally, we address this issue by assessing the percolation transition in the rGO/PDMS composites. The conductivity of the materials for different concentrations of rGO is shown in Fig. 5a. As expected, the conductivity of the composites qualitatively increases with increasing the amount of rGO. However, surprising features are revealed when considering the quantitative data. Indeed, as compared with the pure PDMS matrix, the addition of 1.0 wt% of nanosheets has very little effect on the electrical conduction of the composite by only showing a slight increase (from 2.3 × 10^−10^ to 4.1 × 10^−10^ S m^−1^). Such marginal improvement indicates that the 1.0 wt% sheet loading is well below the percolation threshold. However, at this rGO-PDMS mass ratio, the LC transition has already taken place (Fig. 3c,d), indicating that the flakes with large anisotropy indeed tend to self-assemble into nematic LCs (Fig. 5b) before they form a percolated path along the direction of the conductivity measurement. However, with increasing further the content of flakes up to a critical concentration, the conductivity increases rapidly up to 1.42 × 10^−6^ S m^−1^, which is four orders of magnitude higher than the virgin polymer. In this case, the aligned flakes inside each monodomain are considered to be percolated and eventually form an infinite network of connected particles through the insulating matrix, owing to the fact that each two neighbouring monodomains inherently contact with each other (Fig. 5c). Thus, we confirm the theoretical prediction that the percolation transition occurs at higher concentrations than the orientational ordering transition.

The steep rise of the conductivity of the composite, *σ*_c_, near percolation can be typically described by a bond percolation model^1^ and treated by a power law: 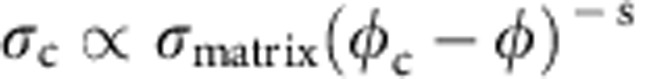, where *σ*_matrix_ is the conductivity of the polymer matrix, *ϕ*_c_ is the onset of connectedness percolation, *ϕ* is the filler content and s is the universal critical exponent in the insulating region. The best fit of the conductivity data to the above power law yields *ϕ*_c_=4.7 wt% (see the inset in Fig. 5a). Such a percolation threshold is much greater than the predicted value (∼0.1 vol%) for randomly oriented oblate ellipsoids (platelets) with an aspect ratio of 1,000, as typically expected for the presently used particles^37^. The common strategy to pursue a low percolation threshold in composites by introducing highly anisotropic particles is shown to fail experimentally for platelets such as graphene nanosheets. However, the high percolation threshold coupled with low conductivity level here indicates that the LC composites can serve as valuable dielectric materials. Indeed, the dielectric constant of composites is expected to diverge at the percolation threshold. Coincidentally, some graphene-based nanocomposites have been shown to be extremely promising as high-*k* materials^12^,^13^,^14^. This feature probably arises from the presently identified tendency of platelets at spontaneously order.

### Dielectric properties of the rGO/PDMS composites

In a percolative polymer composite, as the conducting filler content (*ϕ*) approaches the percolation threshold,*ϕ*_c_, from below, the dielectric constant, *ɛ*_c_, of the composite diverges as 
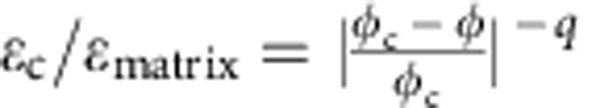
,where *ɛ*_matrix_ is the relative dielectric constant of the polymer matrix and *q* is an exponent of about 1 (ref. 6). This divergent behaviour near percolation is desirable for new high-*k* materials at a low filler loading. However, an intuitive drawback of high-*k* percolative composites is that their high dielectric constant originates from the detailed but sensitive structure of microcapacitors very close to *ϕ*_c_ (ref. 13), which makes the control of such a structure difficult. By contrast, in the present study, the locally aligned graphene flakes in composites can form effective microcapacitors at concentrations much below *ϕ*_c_. The formation of small monodomains of LCs well preserves the microcapacitor structure by largely retarding the percolation transition. As a result, the compositional window for high-*k* performances is considerably expanded.

**Figure 6:**
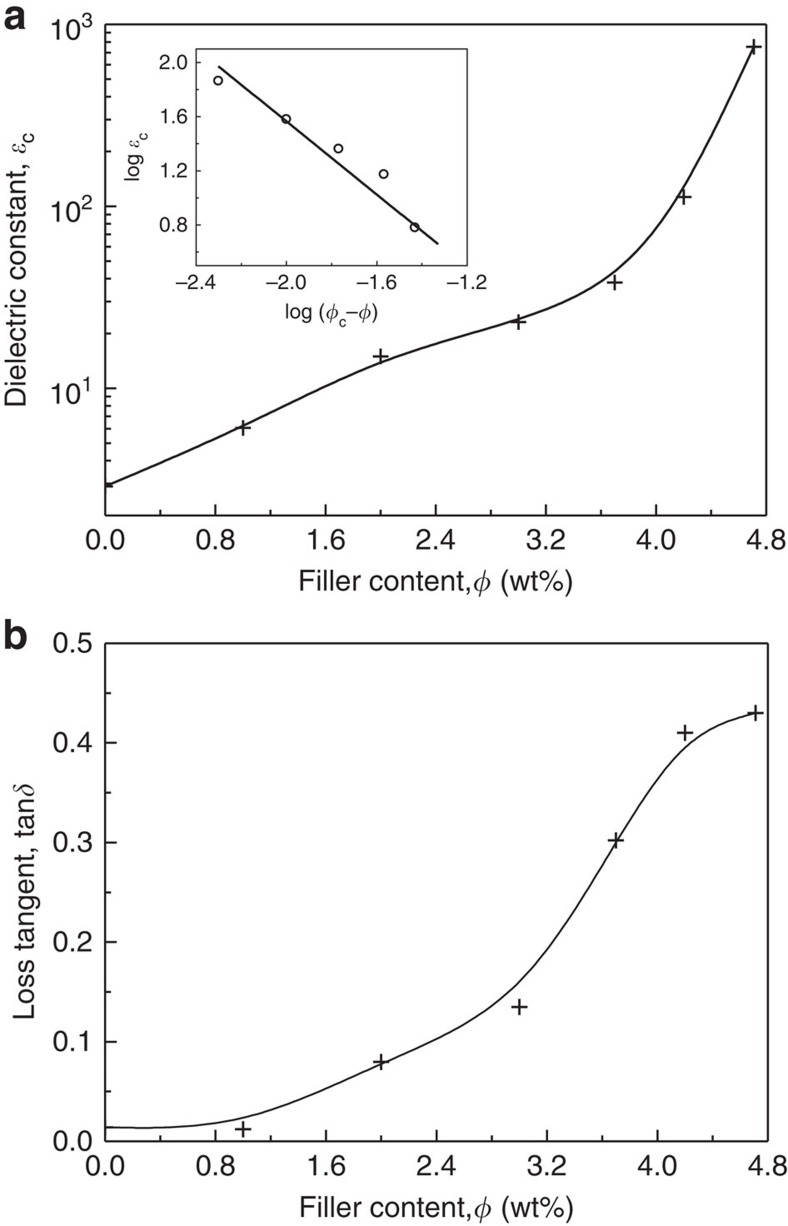
Dielectric properties of the rGO/PDMS composites. (**a**) Dielectric constant of composites, *ɛ*_c_, measured at room temperature and 100 Hz. The inset shows that the dielectric constant can be described by the percolation theory and matches well with the standard model^13^ with *ϕ*_c_=4.7 wt% and *q*=1.15. (**b**) Loss tangent, tan*δ*, of the composites versus filler content, measured at room temperature and 100 Hz.

**Figure 7:**
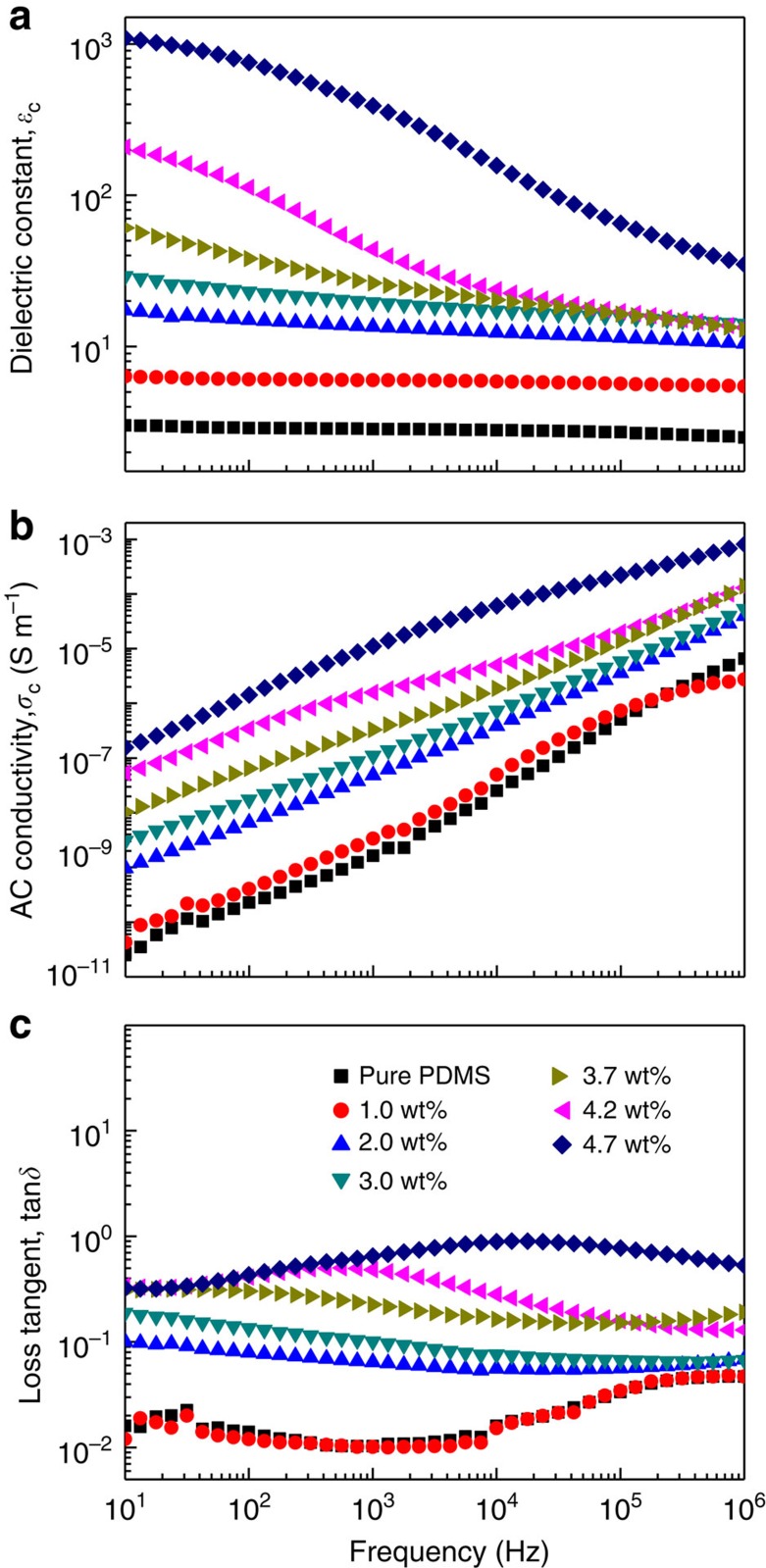
Dielectric properties of rGO/PDMS nanocomposites. The frequency dependence of the dielectric constant, (**a**) AC conductivity (**b**) and loss tangent (**c**) of rGO/PDMS nanocomposites with different weight fractions of rGO.

Figure 6a presents the dielectric constant of the resultant rGO/PDMS composites at varying filler loadings. Variations of the dielectric constant, conductivity and of the loss tangent as a function of frequency are provided in Fig. 7. The dielectric constant at 100 Hz of the composites gradually increases from 2.8 to 23 at low filler contents (<3 wt%), yet dramatically augments to 753 at the percolation threshold, which is 260 times higher than that of pure PDMS. The increase in dielectric constant is ascribed to interfacial polarization as a result of the conductivity contrast between the conducting rGO flakes and the insulating PDMS matrix. Aligned flakes in the monodomains can be viewed as local microcapacitors with the rGO as electrodes and a very thin host polymer layer in between as dielectric (Fig. 5b)^21^. The large rise at percolation results from the growth of the capacitor size as platelets become gradually connected. The average aggregate size diverges, while their separation decreases, explaining thereby a divergence of the dielectric constant at low frequency with the formation of giant capacitors. This general behaviour is in fact qualitatively independent of the nature of the percolating system. However, quantitative features are here optimized with the platelet geometry that favours the formation of capacitors over a large processing window. Indeed, local capacitors are formed already at low concentration because of the shape and structuration of the conductive particles that face each other in the liquid-crystalline state. In addition, their concentration can be increased to concentrations greater than usually expected for anisotropic particles, because the platelets are aligned and therefore less prone to contact each other. As outlined above, it is the LC transition that explains the unusually high percolation threshold of graphene platelets. As a result, the present materials can exhibit a large rise of dielectric constant over a large concentration range, by contrast to other systems in which the near-percolation regime is generally narrow^12^,^13^,^14^. Owing to the unique microcapacitor network structure inside the LC momodomains, the high dielectric performances can be easily tuned in a wide range of filler content between 3.0 and 5 wt%.

Apart from the high dielectric constant, high-*k* materials should also have low loss tangent for realistic applications. Unfortunately, the high dielectric constant in percolative composites is often accompanied by a quite high loss dissipation factor due to the delocalization of charges on a macroscopic scale near percolation as a result of tunnelling or ohmic conduction. For instance, as reported in an isotropic rGO/polyvinylidene fluoride nanocomposite, the loss tangent increased rapidly up to 11 near percolation, owing to the significantly increased conductivity in the nanocomposites^38^. By contrast, in the presently studied composites, due to the formation of LC monodomains, establishing a percolating path of rGO by tunnelling or ohmic conduction becomes more difficult, resulting thereby in a limited conductivity level (Fig. 5a). As a result, as shown in Fig. 6b, the loss tangent undergoes a moderate increase up to only a value of ∼0.43 at 100 Hz.

Lastly, even though the present materials are of primary interest for their dielectric properties, we have also characterized their mechanical properties. A large increase of the Young's modulus is observed with increasing the fraction rGO in the PDMS matrices (Supplementary Fig. 5).

## Discussion

We modified GO flakes by using PAPMS-*b*-PDMS copolymers. The modified GO flakes can be well dispersed in a non-polar organic solvent such as diethyl ether. Nematic LCs formed on increasing the concentration of GO suspensions, which overcomes the practical limitations imposed on liquid-crystalline GO processing in polar liquid media^15^,^17^. A liquid-crystalline graphene-based composite was realized and characterized electrically, to assess the percolation transition. It was found that the graphene flakes with giant anisotropy tend to self-assemble into numerous monodomains of nematic LCs before they form an infinite percolating network. Owing to the LC structures, the composites showed a wide processing window to easily tune high dielectric performances, not as the case of conventional percolative composites in which the dielectric properties are very sensitive to concentration variations near the percolation threshold^5^,^13^,^14^. The wide compositional window coupled with the high dielectric constant and low loss tangent validate the principle that graphene LC retarded percolation provides a route to new and highly efficient high-*k* materials. Although this approach was not clearly identified in earlier studies, it is likely to be that some promising results already established in the development of graphene-based nanocomposites for dielectric applications could be due to the physical mechanisms described in this work. Lastly, even though the present study was performed with conductive nanoplatelets, the concept of competition of connectivity percolation and LC transition does depend only on the shape of the particles and not on their chemical composition or electrical properties. It is therefore expected that a similar behaviour should take place with non-conductive materials such as inorganic clay platelets. Nevertheless, for such materials the competition of LC transition with rigidity percolation, as opposed to connectivity, may be of greater technological interest if mechanical properties are concerned. This problem is still raising questions for future research.

## Methods

### Materials

The monolayer GO aqueous solution is purchased from the Graphenea Company in Spain. It is typically obtained by chemically processing raw graphite material in water. The monolayer content reaches up to 95% and the single layer has a sheet dimension of 2–3 μm. Before use, the received GO solution is diluted to reach a concentration of 1 mg ml^−1^. Three kinds of PAPMS-*b*-PDMS copolymers named copolymer i, ii and iii with 2–3, 4–5 and 6–7 mol% of amine groups, respectively, were purchased from Gelest. Copolymers i, ii and iii have total molecular weights of 4,500–6,000, 7,000–9,000 and 4,000–5,000 g mol^−1^, respectively. The liquid PDMS precursor and curing agent are provided by Dow Corning under the trade name Dow Corning Sylgard 184 silicone elastomer kit. Both the copolymer and PDMS precursors were used as received. The composition of the PDMS precursor and curing agents are provided in Supplementary Note 1.

### Phase transfer

A typical phase transfer process can be described as follows. A certain amount of selected copolymer was first dissolved in diethyl ether and subsequently mixed with the readily diluted GO suspensions (1 mg ml^−1^). Here the volume of diethyl ether used is the same with GO solution. As the diethyl ether is immiscible with water and has a lower density than water, the copolymer solution in diethyl ether would form an upper phase with GO water solution as a bottom phase. This biphasic mixture was magnetically stirred for 12 h so that the modified GO flakes can be recovered in the organic phase as the phase transfer is achieved. Finally, the mixture will separate into two phase, that is, a clear aqueous phase and a thick, brown organic phase. It should be noted that the phase transfer can be well performed even in the presence of PDMS dissolved in the upper organic phase. In this way, the obtained GO/PDMS composite precursor can be used to prepare solid composites based on a typical solution casting method.

### Fabrication of solid composites

To achieve the best solubility of GO in diethyl ether, the copolymer i with 2–3 mol% amine groups was chosen to interact with the GO flakes at a GO/copolymer mass ratio of 1:4. The phase transfer was achieved with polymer matrix PDMS in the upper diethyl ether phase. Afterwards, the collected GO/PDMS composite precursor was used as start materials to prepare solid composites by evaporating solvent and residual water, followed by thermally induced cross-linking with the addition of curing agent. Details of the processes are schematically illustrated in Supplementary Fig. 6 and more information on the composites fabrication is provided in Supplementary Method.

### Characterization

Surface morphologies of the GO flakes before and after copolymer modification were characterized by TEM (Hitachi H600). The Raman spectra were taken using an excitation of wavelength of 532 nm on Horiba Jobin Yvon Xplora. The laser spot size is 1 μm. Samples were prepared by depositing GO aqueous solution or modified GO organic solution on silicon wafers, followed by a completely drying process at room temperature for 12 h. GO and GO/PDMS suspensions (upper phase achieved by phase transfer with and without PDMS dissolved in organic phase, respectively) were taken to investigate their birefringence under a polarizing optical microscope (Leica DM 2500P) with × 10 and × 40 objectives. The morphology of as-prepared GO/PDMS composites was observed by a SEM using a field-emission SEM instrument (JEOL 6700FEG) after sputter coating with platinum. The GO and rGO-based PDMS composites are further examined under a TEM (Hitachi H7650). For TEM analysis, the composites were ultramicrotomed to slices of 40–60 nm thickness (Leica UC7). X-ray data were collected on a Rigaku Nanoviewer (XRF microsource generator, MicroMax 007HF), with a 1,200-W rotating anode coupled to a confocal Max-Flux Osmic mirror (Applied Rigaku Technologies, Austin, USA) and a MAR345 image plate detector (MARResearch, Norderstedt, Germany). Samples were put in quartz capillaries, which are exposed to the incident X-Ray beam. The detector is placed at a distance of 1,193 mm, providing access to 2*θ* angle in the 0.15°–4° range (0.1–3 nm^−1^). The dielectric properties of the rGO/PDMS composites were characterized as a function of frequency (0.1–10^6^ Hz) at room temperature using an impedance analyser (7260 Impedance Analyzer, MaterialsMates Italia). Before measurement, silver grease has been applied on the sample surfaces for contacting.

## Additional information

**How to cite this article:** Yuan, J. *et al*. Graphene liquid crystal retarded percolation for new high-*k* materials. *Nat. Commun.* 6:8700 doi: 10.1038/ncomms9700 (2015).

## Supplementary Material

Supplementary Information Supplementary Figures 1-6, Supplementary Note 1, Supplementary Discussion, Supplementary Method and Supplementary References
