# Supplementary material for: Graphene liquid crystal retarded percolation for new high-k materials
Source: Nat Commun. 2015 Nov 16;6:8700. doi: 10.1038/ncomms9700 (PMC4660061; doi:10.1038/ncomms9700)
Supplement: Supplementary Information — Supplementary Figures 1-6, Supplementary Note 1, Supplementary Discussion, Supplementary Method and Supplementary References [file ncomms9700-s1.pdf]

Supplementary Figures

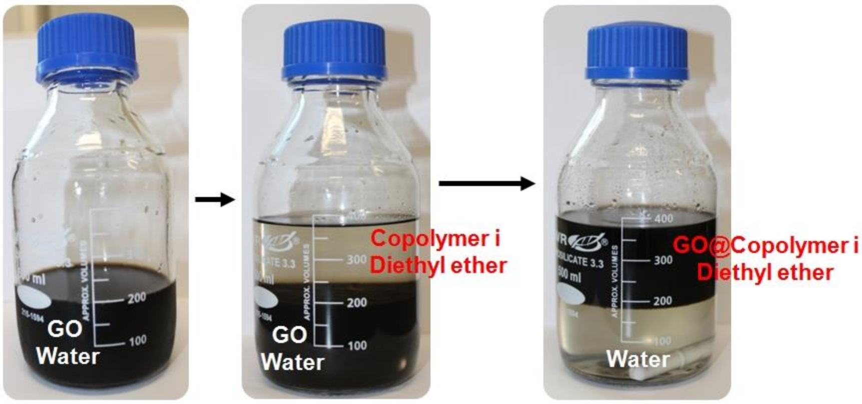

Supplementary Figure 1 | Phase transfer with GO aqueous solution (1mg/ml). The phase transfer can be easily scaled up to large volumes of biphasic mixtures. 0.2 g GO flakes can be completely extracted from 200 ml GO aqueous solution (1 mg/ml) to organic diethyl ether phase by using 0.8g copolymer i. Larger volumes and phase extraction methods, including industrial and continuous processes, could be used similarly

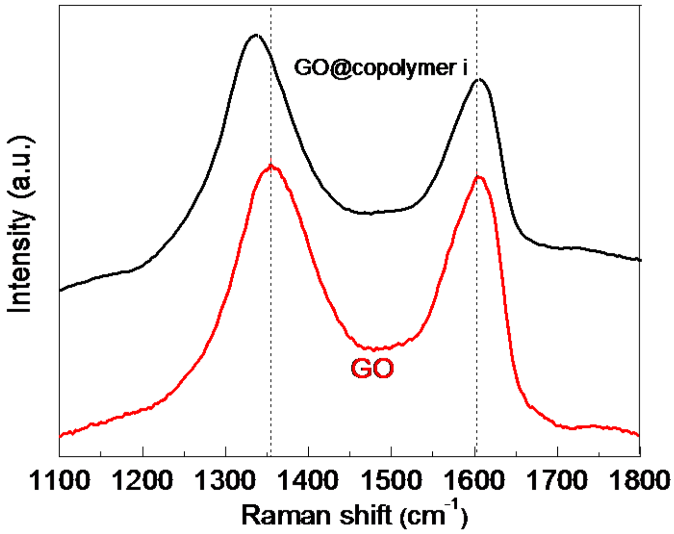

Supplementary Figure 2 | Raman spectra of pristine GO and GO@copolymer i flakes. The spectra were taken using an excitation of wavelength of 532 nm. Samples were prepared by depositing GO aqueous solution or GO@copolymer organic solution on silicon wafers, followed by a completely drying process at room temperature for 12 hours.

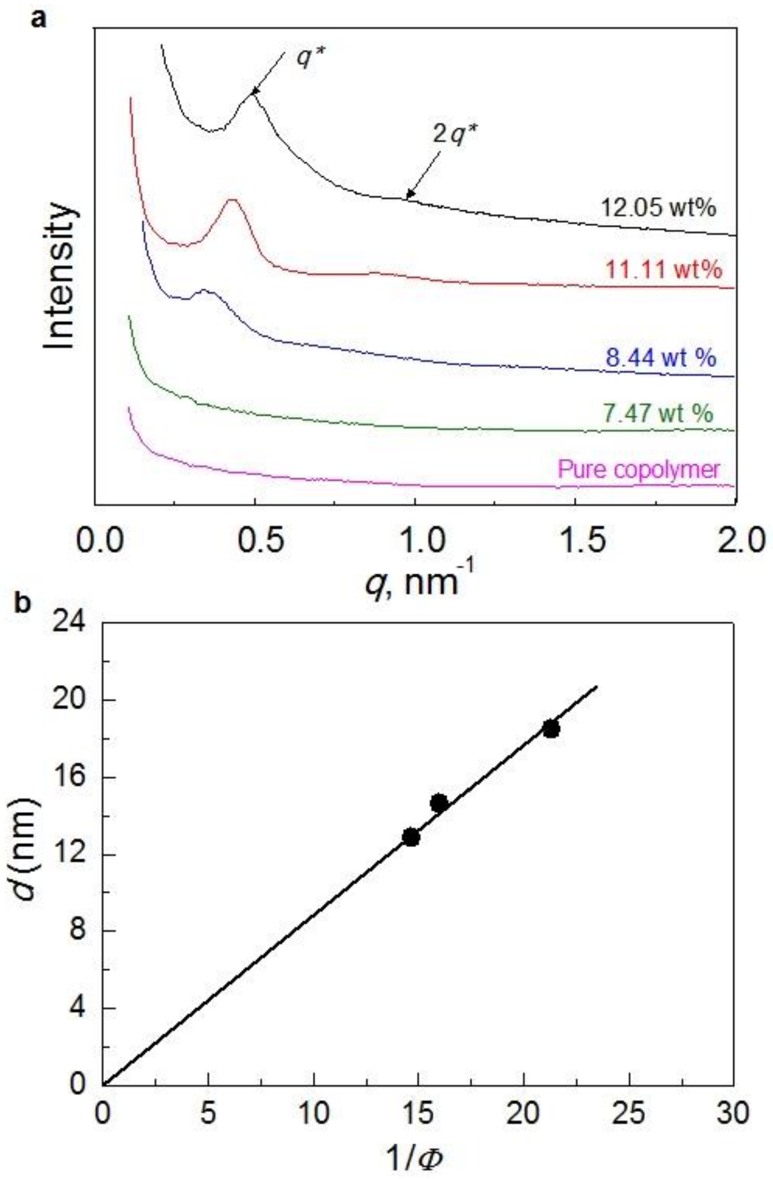

Supplementary Figure 3│SAXS characterizations of GO liquid crystals in PAPMS-*b*-PDMS copolymer. Spectra as a function of the scattering wave vectors for different weight fractions of GO (**a**). First order diffraction peaks are clearly observed at q^* for the most concentrated materials. *d* spacing between GO sheets as a function of $1/\phi$ (**b**). The black line is linear fit that allows the effective thickness of the sheets to be determined.

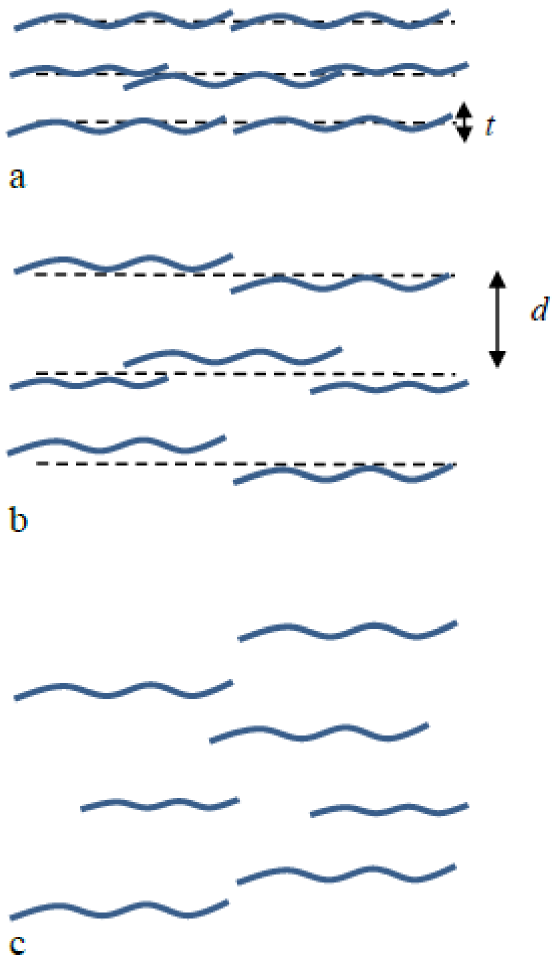

Supplementary Figure 4 | Evolution of the GO liquid crystalline structure. Schematic of the evolution of the structure of GO liquid crystals in PAPMS-*b*-PDMS with decreasing concentration from (**a**) to (**c**). Orientational order is maintained upon dilution but spatial correlations become less pronounced as the material is diluted. This is reflected by the disappearance of diffraction peaks in the SAXS spectra of more dilute samples.

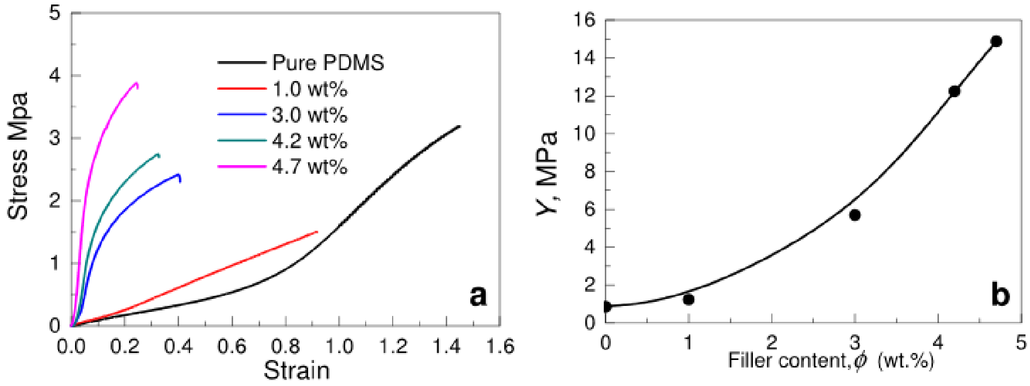

Supplementary Figure 5 | Mechanical properties of rGO/PDMS composites. Stress *vs* strain curves of the materials for different fractions of rGO (**a**). Young's modulus as a function of the weight fraction of the filler (**b**).

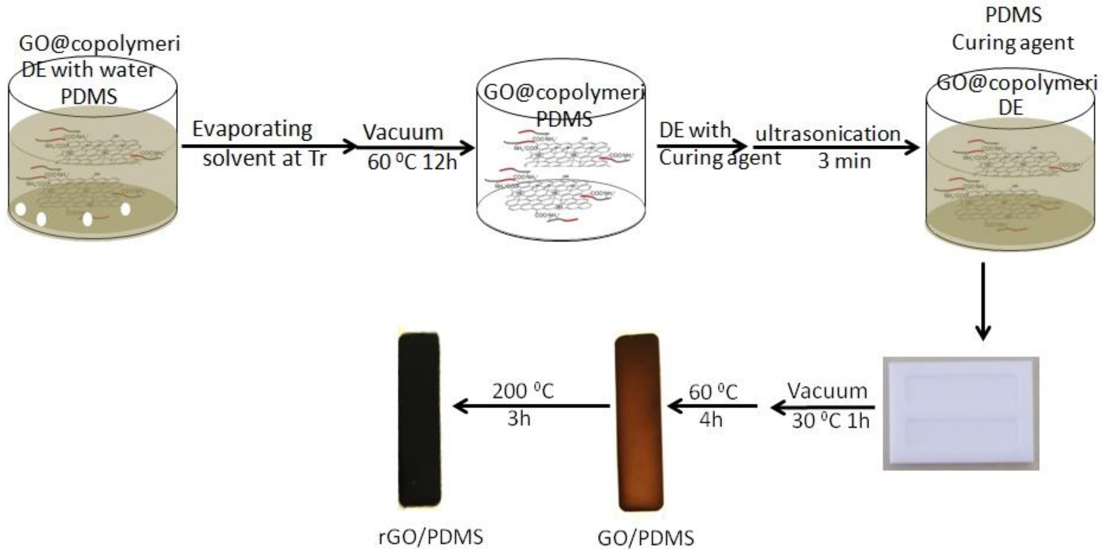

Supplementary Figure 6 | The protocol used to prepare composites. The GO- and rGO-based PDMS composites are prepared by starting from the obtained GO/PDMS composite precursor. Here DE is the abbreviation of diethyl ether.

Supplementary Note1:

The liquid PDMS precursor and curing agent are provided by Dow Corning under the trade name Dow Corning Sylgard 184 silicone elastomer kit. According to the provider, the base part of the kit contains the following PDMS precursors:

1. Dimethyl siloxane, dimethylivinyl terminated
2. Dimethylvinylated and trimethylated silica
3. Tetra (trimethoxysiloxy) silane
4. Ethyl benzene

The base contains 8 grams/liter of volatile organic compounds.

The curing agent contains the followings:

1. Dimethyl, methylhydrogen siloxane
2. Dimethyl siloxane, dimethylvinyl terminated
3. Dimethylvinylated and trimethylated silica
4. Tetramethyl tetravinyl cyclotetra siloxane
5. Ethyl benzene

Supplementary Discussion

Supplementary **Figure 2** shows the Raman spectra in a range of 1100-1800 cm^{-1} for pristine GO and GO@copolymer flakes. As expected, pristine GO has two characteristic peaks localized at 1355 cm^{-1} and 1601 cm^{-1}, which are respectively assigned as the D and G bands.[1] The G band arises primarily from first-order scattering of the E_{2g} vibrational mode of sp^2-hybridized carbon atoms, while the D band originates from the vibrations of carbon atoms with dangling bonds of

disordered graphite. It corresponds to the presence of point defects, residual oxygen as well as the structural disorder induced by folds, wrinkles, and tears in GO flakes.[2] After functionalization with the copolymer i, the G band was found unchanged but the D band was blue-shifted at 1335 cm^{-1}, indicating that the copolymer did not modify the sp^2 carbon lattice structure with only interacting with functional groups of GO sheets (defects).

Liquid crystals of GO in PAPMS-*b*-PDMS have been characterized by Small Angle X-ray Scattering (SAXS). X-ray scattering spectra of samples with different weight fractions of GO are shown in supplementary **Fig. 3(a)**. The more concentrated materials exhibit a well-defined first order diffraction peak at a wave vector q^*. A second order diffraction peak at $2q^*$ is observed for materials at 12.05wt% and 11.11wt%. Such an ordering is typical of a lamellar phase and the present discotic nematics can be considered as pseudo-smectic liquid crystals.[3] However, pseudo-smectic ordering is lost when the concentration is decreased. The first order peak even disappears for concentrations below 6wt%. This behavior reflects a loss of positional ordering as the materials are diluted. The typical evolution of the structure upon dilution is sketched in supplementary **Fig. 4**. Nevertheless, the most concentrated materials still allow the effective thickness t of the platelets, and the d spacing between the platelets, with $d=2\pi/q^*$, to be determined. Indeed, in a monophasic system in which the platelets repel each other, d and t are expected to vary proportionally according to $d=t/\phi$.[3] As shown in supplementary **Fig. 3(b)**, such a linear relationship is actually observed; confirming thereby that the platelets are well

stabilized by steric repulsions provided by adsorbed PAPMS-*b*-PDMS copolymers. The effective thickness of the platelets determined from the above behavior is found to be of about 0.85nm considering a density of 1.8 g/ml for GO materials, and of 0.96 g/ml for PAPMS-*b*-PDMS copolymer.

The presence of first order and even second order diffractions peaks in SAXS spectra of concentrated liquid crystals indicate strong spatial correlations between the flakes. The latter form a discotic nematic phase which can be viewed as a pseudo-smectic phase. The *d* spacing between the flakes increases upon dilution. This behavior results from the repulsive interactions that the flakes experience in the PAPMS-*b*-PDMS fluid. It allows the effective thickness *t* between the flakes to be measured (supplementary **Fig. 3(a)**). However, no peaks are observed when the samples become more diluted, in spite of their well maintained nematic ordering reflected by their strong optical birefringence. This behavior indicates that spatial correlations become less pronounced whereas orientational ordering is still well preserved. The evolution of the structure of the present liquid crystals is sketched in supplementary **Fig. 4**.

The mechanical properties of reduced graphene oxide-PDMS composites have been characterized under tensile load using a Zwick Roell testing machine equipped with a 100 N load cell. The stress *vs* strain curves of composites with different weight fraction of rGO are shown in supplementary **Fig. 5**. It is observed that the Young's modulus of the materials increases with increasing the amount of rGO filler. This increase of stiffness is associated to a reduction of the strain at break.

Supplementary method

Liquid crystallinity of GO has been established for several years in aqueous media [3-9] and polar organic solvents [10-13]. But these materials often contain ions which are easily dissociated because of the high dielectric constant of the suspending medium. In particular GO materials in such media remain stabilized by electrostatic interactions specifically due to ion dissociation in contrast to the stabilization in nonpolar solvents which is provided by steric repulsion. Dissociated ions in polar media not only introduce large conductivity losses but also limit the maximal voltage that can be applied on such liquid crystals. Materials processed in nonpolar media are less prone to contain dissociated ions and can therefore be more suitable for applications as high-*k* materials.

In spite of the demonstrated versatility of GO liquid crystals, routes towards solid composites through thermally equilibrated states actually have not yet been established from aqueous or polar solvent solutions to date. Indeed solid materials are usually achieved by rapid solvent evaporation,[14] fiber wet-spinning[12] or other flow induced assembly [15-17] mechanisms. By contrast to these approaches, the present processing in a nonpolar solvent allows a direct achievement of solid composites by using liquid, polymerizable and cross-linkable precursors as suspending medium without any flow or volume changes during solidification. The detailed procedures are presented in **Fig. 6**.

Starting from the collected GO/PDMS composite precursor, one can prepare solid composites by evaporating solvent and residual water followed by thermally induced

cross-linking with addition of curing agent, The solvent was first evaporated at room temperature to obtain a viscous GO/PDMS fluid. This fluid was further dried at 60 °C for 12 h in the vacuum oven to completely remove the residual solvent and water. The applied low drying temperature is intended to avoid the thermal reduction of GO at this stage which may lead to the restacking of GO flakes. Because of the very high viscosity of the obtained GO/PDMS mixture, a small amount (4 ml) of solvent diethyl ether can be added into the 1g of GO/PDMS mixture to lower the viscosity and facilitate further processing. A curing agent was added into this liquid composite with a ratio of 1:10 relative to the PDMS base. A short tip sonication (less than 3 min) was applied on this GO/PDMS mixture to homogenize the dispersions of the curing agent in the solvent. Then the GO/PDMS mixture was molded, degassed for 1 h and cured at 60 °C for 4 h. Finally in-*situ* thermal reduction of GO was performed in the solid composites to increase their conductivity without changing the nanosheet dispersion state in the final material.

Supplementary References

1. Kudin, K.N. *et al.* Raman spectra of graphite oxide and functionalized graphene sheets. *Nano Lett.* **8**, 36-41 (2008).
2. Eda, G., Fanchini, G. & Chhowalla, M. Large-area ultrathin films of reduced graphene oxide as a transparent and flexible electronic material. *Nat. Nanotechnol.* **3**, 270-274 (2008).
3. Zamora-Ledezma, C. *et al.* Liquid crystallinity and dimensions of surfactant-stabilized sheets of reduced graphene oxide. *J. Phys. Chem. Lett.* **3**, 2425-2430 (2012).
4. Aboutalebi, S.H., Gudarzi, M.M., Zheng, Q.B. & Kim, J.-K. Spontaneous formation of liquid crystals in ultralarge graphene oxide dispersions. *Adv. Funct. Mater.* **21**, 2978-2988 (2011).

5. Dan, B. *et al.* Liquid crystals of aqueous, giant graphene oxide flakes. *Soft Matter* **7**, 11154-11159 (2011).
6. Hu, X., Xu, Z., Liu, Z. & Gao, C. Liquid crystal self-templating approach to ultrastrong and tough biomimic composites. *Sci. Rep.* **3**, 2374 (2013).
7. Jalili, R. *et al.* Scalable one-step wet-spinning of graphene fibers and yarns from liquid crystalline dispersions of graphene oxide: Towards multifunctional textiles. *Adv.Funct. Mater.* **23**, 5345-5354 (2013).
8. Kim, J.E. *et al.* Graphene oxide liquid crystals. *Angew. Chem. Int. Ed.* **50**, 3043-3047 (2011).
9. Xu, Z. & Gao, C. Aqueous liquid crystals of graphene oxide. *Acs Nano* **5**, 2908-2915 (2011).
10. Jalili, R. et al. Organic solvent-based graphene oxide liquid crystals: A facile route toward the next generation of self-assembled layer-by-layer multifunctional 3D architectures. *Acs Nano* **7**, 3981-3990 (2013).
11. Behabtu, N. *et al.* Spontaneous high-concentration dispersions and liquid crystals of graphene. *Nat.Nanotechnol.* **5**, 406-411 (2010).
12. Seyedin, M.Z., Razal, J.M., Innis, P.C., Jalili, R. & Wallace, G.G. Achieving outstanding mechanical performance in reinforced elastomeric composite fibers using large sheets of graphene oxide. *Adv. Funct. Mater.* **25**, 94-104 (2015).
13. Gudarzi, M.M., Moghadam, M.H.M. & Sharif, F. Spontaneous exfoliation of graphite oxide in polar aprotic solvents as the route to produce graphene oxide - organic solvents liquid crystals. *Carbon* **64**, 403-415 (2013).
14. Yousefi, N. *et al.* Simultaneous in situ reduction, self-alignment and covalent bonding in graphene oxide/epoxy composites. *Carbon* **59**, 406-417 (2013).
15. Naficy, S. *et al.* Graphene oxide dispersions: tuning rheology to enable fabrication. *Mater. Horiz.* **1**, 326-331 (2014).
16. Liu, Z. *et al.* Wet-spun continuous graphene films. *Chem. Mater.* **26**, 6786-6795 (2014).
17. Park, S. *et al.* Graphene oxide papers modified by divalent ions - Enhancing mechanical properties via chemical cross-linking. *Acs Nano* **2**, 572-578 (2008).
